# Probe reconstruction for holographic X-ray imaging

**DOI:** 10.1107/S160057751700128X

**Published:** 2017-02-16

**Authors:** Johannes Hagemann, Anna-Lena Robisch, Markus Osterhoff, Tim Salditt

**Affiliations:** a Institut für Röntgenphysik, Friedrich-Hund-Platz 1, 37077 Göttingen, Germany

**Keywords:** X-ray near-field imaging, ptychography, probe characterization

## Abstract

In X-ray holographic near-field imaging the resolution and image quality depend sensitively on the beam. Artifacts are often encountered due to the strong focusing required to reach high resolution. Here, two schemes for reconstructing the complex-valued and extended wavefront of X-ray nano-probes, primarily in the planes relevant for imaging (*i.e.* focus, sample and detection plane), are presented and compared. Firstly, near-field ptychography is used, based on scanning a test pattern laterally as well as longitudinally along the optical axis. Secondly, any test pattern is dispensed of and the wavefront reconstructed only from data recorded for different longitudinal translations of the detector. For this purpose, an optimized multi-plane projection algorithm is presented, which can cope with the numerically very challenging setting of a divergent wavefront emanating from a hard X-ray nanoprobe. The results of both schemes are in very good agreement. The probe retrieval can be used as a tool for optics alignment, in particular at X-ray nanoprobe beamlines. Combining probe retrieval and object reconstruction is also shown to improve the image quality of holographic near-field imaging.

## Abstract 2

A comparison of different schemes for probe characterization of an X-ray nano-probe in a near-field imaging setting is presented.

## 1. Introduction   

Preparation of the X-ray probe for coherent imaging applications is indispensable in order to reach high resolution and quantitative contrast. This includes control of focusing, coherence and wavefront. A particular case in point is the quasi-spherical wavefronts required for holographic full-field tomography (Mokso *et al.*, 2007 ▸; Krenkel *et al.*, 2015 ▸; Bartels *et al.*, 2015*a* ▸). In these high-resolution experiments, propagation images are recorded in a divergent beam to achieve the required magnification. Hence nano-focusing (Stangl *et al.*, 2013 ▸) is required, even though the sample is placed in the defocus plane located several millimetres to centimetres behind the focal plane. In order to process the raw images in propagation imaging, before phase retrieval is applied, idealizing assumptions are made with regard to the beam, such as point-source emission or distortion-free wavefront. The validity of such assumptions has recently been investigated, showing that they lead to reduced resolution and image quality (Homann *et al.*, 2015 ▸; Hagemann *et al.*, 2014 ▸). Aiming at more appropriate schemes for treating the data, recent work has introduced the concept of simultaneous reconstruction of probe and object to near-field (propagation) imaging (Stockmar *et al.*, 2013 ▸; Robisch *et al.*, 2015 ▸). This was achieved by a suitable generalization and extension of the ptychographic algorithms initially developed for confined beams (typical for far-field diffractive imaging) (Maiden & Rodenburg, 2009 ▸; Thibault *et al.*, 2009 ▸; Schropp *et al.*, 2010 ▸; Kewish *et al.*, 2010 ▸; Vine *et al.*, 2009 ▸; Marchesini *et al.*, 2016 ▸). Stockmar *et al.* (2013 ▸, 2015 ▸) scanned the object transversally in the extended wavefield behind a wavefront diffuser, in order to increase the diversity of the probe. Thus the wavefront was modified, and hence the ‘natural’ probe was not recovered. Contrarily, Robisch *et al.* (2015 ▸) used the diversity generated by lateral and longitudinal shifts of the object in the beam to recover the natural probe of the setup along with the object. Since beam reconstruction in one plane gives access to the wavefront in all other planes, based on numerical propagation, one may wonder why a near-field reconstruction is needed at all.

For the last few years, X-ray nano-focus optics have been characterized by far-field ptychographic means, scanning an object in or near the focal plane (see, for example, Kewish *et al.*, 2010 ▸; Schropp *et al.*, 2010 ▸). While this is correct in principle, we show in this study that the small distortions in the probe which significantly hamper the image quality of full-field imaging can only be properly ‘probed’ in the defocus plane. Since the mirror height deviations are almost atomically flat, the focal field distribution differs only in the extreme tails up to 10 µm in the focal plane of the probe from the ideal intensity distribution. Therefore, probe reconstruction from far-field data measured with a detector with large pixel size, *e.g.* 172 µm for a Pilatus (DECTRIS) detector, does not cover the field of view (FOV) in the focal plane to include the tails. Thus the propagation of such a reconstructed probe in the near-field does not accurately account for the characteristic fringes of the KB pattern, as measured with a high-resolution detector in the near-field. Contrarily, near-field probe retrieval is perfectly able to accomplish this. To this end, we propose the following: if you measure in the defocal plane, reconstruct in the defocal plane.

At the same time, we are interested in a complete characterization which also includes the field in the focal plane. This was previously not accessible, since in the data of Stockmar *et al.* (2013 ▸) and Robisch *et al.* (2015 ▸) the FOV is cut out from the central part of the probe. This is not sufficient to obtain complete information on the probe, *i.e.* it is, for example, not possible to reconstruct the size of the focal spot, which is obviously essential information for the maximum achievable resolution. In this work we record the complete decay of the probe at the holography end-station GINIX (Göttingen Instrument for Nano-Imaging with X-rays) (Salditt *et al.*, 2015 ▸; Kalbfleisch, 2012 ▸) at the P10 beamline of Petra III (DESY, Hamburg), and use it for reconstruction based on an improved multiple magnitude projections (MMP) scheme (Allen & Oxley, 2001 ▸; Hagemann *et al.*, 2014 ▸; Loetgering *et al.*, 2015 ▸) as well as the near-field ptychography (NFP) scheme (Robisch *et al.*, 2015 ▸). We recorded two independent data sets: one for NFP with the aforementioned lateral and longitudinal shifts of an object with a fixed focus-to-detector distance; the other set for MMP consisting of a detector scan along the longitudinal direction, *i.e.* the focus-to-detector distance is varied. The fundamental difference in these data sets (in view of probe reconstruction) is the way the data diversity is introduced. In NFP, a mixing operation is performed of probe *P* and object *O*, while for MMP the changes in the distance of the detection plane introduce diversity. MMP can not only be exploited for probe reconstruction but also for object reconstruction, as demonstrated before in other wavelength regimes (Allen & Oxley, 2001 ▸; Loetgering *et al.*, 2015 ▸). The two independent approaches yield probe reconstructions which are in very good agreement. Beyond reconstruction of the probe, the presented scheme bears significant advantages also for imaging, *i.e.* reconstruction of objects. Note that most alternative phase-retrieval algorithms in the near-field setting, which also exploit longitudinal scanning (diversity) such as the contrast transfer function reconstruction (Cloetens *et al.*, 1999 ▸) or the transport of intensity equation (Gureyev & Nugent, 1997 ▸; Krenkel *et al.*, 2013 ▸), rely on assumptions (pure phase object, slowly varying phase, linearity of the propagation) of the wavefield under reconstruction, which limit the range of their applicability. None of these restrictions apply to the MMP or NFP schemes.

§2 introduces the experimental setup and the measurement schemes. In §3 an optimized version of the MMP algorithm is introduced, suitable for diverging beams and noisy data. §4 compares the wavefield reconstructions of NFP and MMP, both in and around the focal plane and the far-field. We close the paper in §5 with some practical considerations on how the presented methods can be used for nano-focus optimization and alignment.

## 2. Experimental setup   

**Figure 1:**
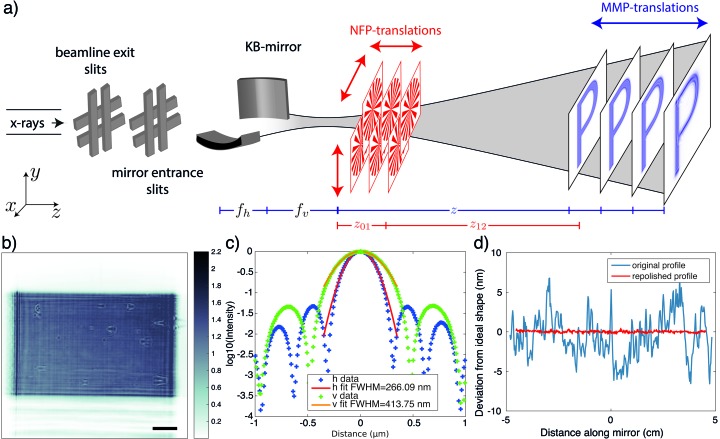
Experimental setup. (*a*) Basic sketch of the nano-focus instrument (GINIX setup at P10 beamline PETRAIII) and data acquisition scheme. The monochromatic beam is focused by a KB mirror system, placed 88 m upstream from the undulator source. The beam size in front of the KB is controlled by two pairs of slits. For the MMP scheme, the empty beam intensity distribution, represented by a ‘P’, is recorded at different defocus positions *z* (blue translations) along the optical axis with no additional object in the beam path. Contrarily, the NFP scheme (red translations) requires an additional test object placed (Siemens star) at varied defocus positions 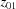 and an overlapping scan in the transversal direction, while the detector distance is fixed. (*b*) Example of the beam intensity distribution recorded for 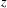 = 0.3346 m for a 400 µm × 400 µm slit opening. Scale bar: 100 µm. (*c*) Intensity distribution along the principle axis (horizontal, vertical) in the focal plane, as simulated numerically by a Huygens principle approach (Osterhoff & Salditt, 2011 ▸) for a 100 µm × 100 µm slit setting, and the measured height profile of the mirrors. (*d*) Deviations from the ideal height profile for the vertical focusing mirror, shown for the original and upgraded mirror.

The experiment was carried out at the nano-focus end-station (GINIX) of the P10 undulator beamline (Salditt *et al.*, 2015 ▸) with photon energy set to 8 keV by a Si(111) channel-cut monochromator. Fig. 1(*a*) ▸ shows a sketch of the setup. A set of slits allowed the illuminated area of the Kirkpatrick–Baez (KB) mirrors to be controlled, and hence also the divergence of the focused beam. The different measurement schemes are colour-coded in red (NFP) and blue (MMP). The intensity patterns were recorded by a scintillator (LUAG) coupled CCD (PCO pco.2000) with 20× magnification microscope lens, resulting in an effective pixel size of 370 nm. The detector was placed on a motorized stage following the beam’s optical axis. For MMP, empty beam recordings were acquired at four detector defocus distances of *z* = {0.3643, 0.3542, 0.3443, 0.3346 m} with an exposure time of 0.1 s. This series of measurements was obtained for the following settings of the beamline slits: 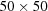, 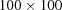, 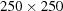, 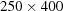, 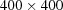 [horizontal (µm) × vertical (µm)]. A typical measurement is shown in Fig. 1(*b*) ▸. For NFP, an additional object is required. Here we used a Siemens star test pattern with 100 nm thickness of tantalum (NTT-AT). The object was placed at different defocus distances 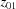 (see Table 1 ▸). At each distance, a lateral scan with step size of 5 µm of 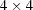 points was performed with 40 ms exposure time. The detector was kept at a fixed position at 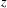 = 0.3723 m. The KB mirrors have been recently upgraded by state-of-the-art elastic emission machining (EEM) polishing (Yamauchi *et al.*, 2002 ▸), resulting in a height deviation (from the ideal ellipse, peak to valley) of 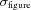 = 0.89 nm, 0.88 nm and a root-mean-square roughness 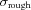 = 0.09 nm, 0.1 nm, for the horizontal (h) and the vertical (v) mirror, respectively [see height profile function in Fig. 1(*d*) ▸].

This corresponds to a 5.4 (h), 15.8 (v)-fold improvement for the figure errors and a 4.4 (h), 1.5 (v)-fold improvement for the roughness over the initial values (Kalbfleisch, 2012 ▸; Salditt *et al.*, 2015 ▸). Note that for these near atomically flat reflecting surfaces the focal intensity distribution becomes almost identical to the ideal case, over four orders of magnitude in the intensity, as shown in Fig. 1(*c*) ▸. Contrarily, the flat-field pattern still shows the characteristic stripes originating from the height deviations.

## 3. MMP algorithm   

Reconstruction of a nano-focus probe *P* amounts to the reconstruction of a complex-valued wavefield Ψ from intensity measurements, *i.e.* it is a perfect example of solving the phase problem. In comparison with Hagemann *et al.* (2014 ▸), we use here an optimized algorithmic approach for MMP based on Luke (2005 ▸), which we will call sequential relaxed averaged alternating reflections (sRAAR), since the projection on the measurements is carried out in a sequential manner. An iteration of sRAAR is given by 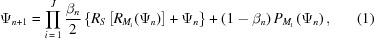
where 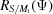 = 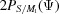 − 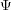 denotes a (mirror) reflection by a given constraint set, and 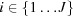 enumerates the intensity measurements 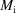. *J* influences the accuracy of the reconstruction; already for 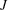 = 2, given that the change in the Fresnel number is sufficient, we can obtain reconstructions for Ψ. Increasing *J* further increases the accuracy, but at the expense of more costly numerical operations. The parameter 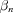 controls the relaxation, and is varied as a function of the iteration number *n* according to 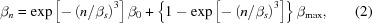
where 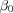 denotes the starting value, 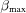 the final value of 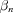, and 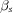 the iteration number when the relaxation is switched. This relaxation strategy follows equation (37) of Luke (2005 ▸). A value of 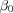 close to 1 helps in the beginning to efficiently sample the possible solutions; during the later iterations the smaller 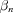 helps to draw the weight on the measurements. The projection on the measurements 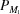 is given by
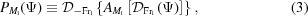
where 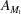 is the actual adaptation of amplitudes, given by 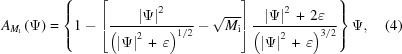
which follows Luke *et al.* (2002 ▸), where ∊ is a constant to prevent a division by 0 in the order of magnitude of the machine precision. Note that this implementation of the projection on the measurement constraint introduces a smooth perturbation, which improves numerical stability (Luke, 2005 ▸). The propagation to the individual measurement planes is performed by the Fresnel propagator 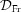, for a given Fresnel number 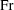 = 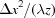 with respect to the pixel size 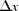, 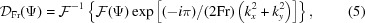
where 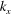 and 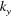 are frequencies in Fourier space.

The operator 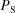 applies a support constraint in the focal plane, given by
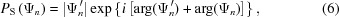
where 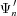 is given by 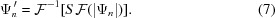
This is basically a back propagation to the focal plane neglecting the curvature followed by application of a support constraint. The support constraint *S* is defined as
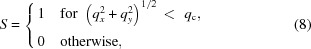
where 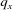 and 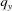 denote coordinates in the focal plane and 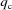 is the cut-off value. The hard cut-off can be relaxed by using a Gaussian window. Applying *S* directly on 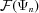 leads to a propagation by a unknown distance, since the curvature is not exactly known in the beginning of the reconstruction process. This problem is circumvented by taking the modulus. The algorithm and the projectors in use have been tested in a numerical experiment; for details refer to the supporting information.

## 4. Results   

**Figure 2:**
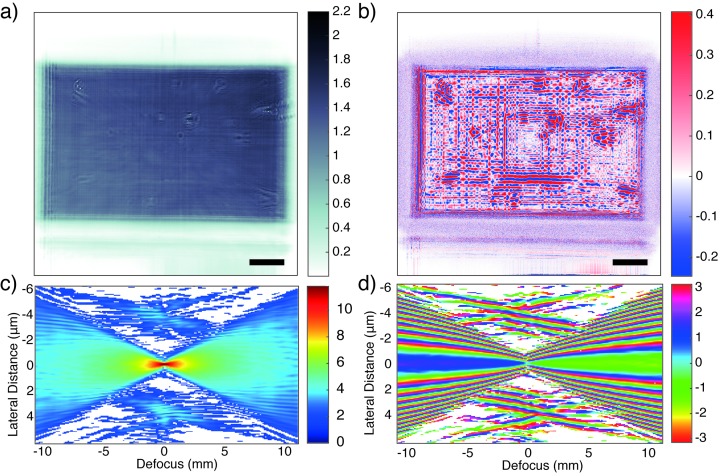
Probe reconstructed from MMP for the 400 µm × 400 µm slit setting, showing (*a*) amplitude and (*b*) phase, in the detection plane at 0.3643 m. Scale bar: 100 µm. The corresponding propagation profile is shown in (*c*) for intensity (logarithmic) and (*d*) for phase along the optical axis ±10 mm around the focal plane in the vertical direction. Pixels with intensity values smaller than 10^−5^ of the maximum value were masked out in white.

The MMP algorithm described in §3 was applied to the data, after performing the following raw data processing steps. After subtraction of a dark image the intensities were scaled to mean amplitude 1 and then aligned to the centre of mass of the contour of the beam. Other alignment schemes like Fourier space registration (Guizar-Sicairos *et al.*, 2008 ▸) do not work for this kind of data, due to the divergence. The pixel size of all distances has been reduced by interpolation to 37 nm. This high sampling is necessary for artifact-free Fresnel propagation, in particular to account for the rapidly varying chirp functions of the spherical contribution of the phase, otherwise the propagation in between the measurement planes is inconsistent. Note that in contrast to many previous treatments and the NFP implementation below, the MMP data are not transformed to an equivalent parallel beam geometry (by Fresnel scaling theorem), but treated in the direct coordinate system. After preprocessing, the data were used as input for sRAAR presented in §3. The parameters for sRAAR were 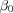 = 0.99, 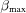 = 0.75 and 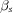 = 150. sRAAR was iterated 2000 times starting from the measured amplitudes in the plane at 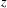 = 0.3643 m multiplied by the phases of a Gaussian beam, giving a first guess for the curved wavefront. For the reconstructions we assumed 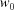 = 250 nm, where 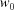 is the waist of a Gaussian beam. We chose for the support constraint 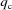 = 200*w*_0_. Fig. 2 ▸ shows the result for a typical imaging configuration of the exit slits with 400 µm × 400 µm. In this configuration the mirrors are fully illuminated, *i.e.* the maximal length of the mirrors is illuminated. This correspondingly highest numerical aperture results in the smallest focal width of (192 ± 2) nm × (170 ± 1) nm (h × v), as determined from the reconstructed focus *via* fitting a Gaussian function with linear background. The reconstructed probe wavefield is shown in Figs. 2(*a*) (amplitude) and 2(*b*) ▸ (phase) at the detection plane at 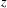 = 0.3643 m. Assuming that the far-field approximation holds, which is well justified in view of the small Fresnel number (Fr = 2.5 × 10^−5^), we apply the Fourier transform to recover the probe in the focal plane. By application of the Fresnel propagator, we can then simulate the propagation around the focus [see Figs. 2(*c*) and 2(*d*) ▸].

**Figure 3:**
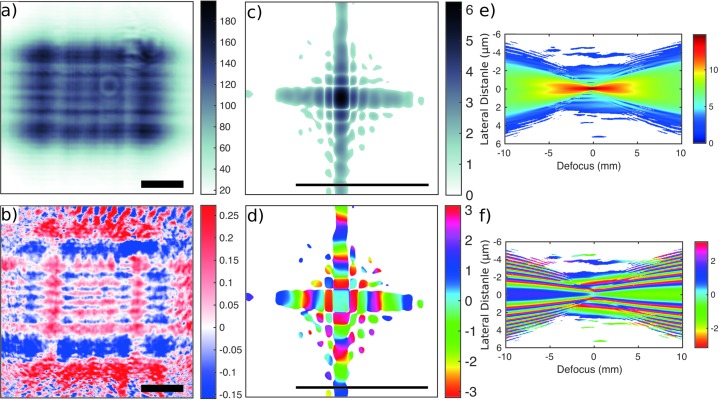
Probe reconstructed from NFP for the 100 µm × 100 µm slit setting, showing (*a*) amplitude and (*b*) phase in the detection plane at 0.3732 m. Scale bar: 50 µm. The focus intensity (*c*) and phase (*d*) are obtained by Fourier transformation. Scale bar: 5 mm. The corresponding propagation profile is also shown in (*e*) for intensity (logarithmic) and (*f*) for phase along the optical axis ±10 mm around the focal plane in the vertical direction. Pixels with intensity values smaller than 10^−5^ of the maximum value were masked out in white.

**Figure 4:**
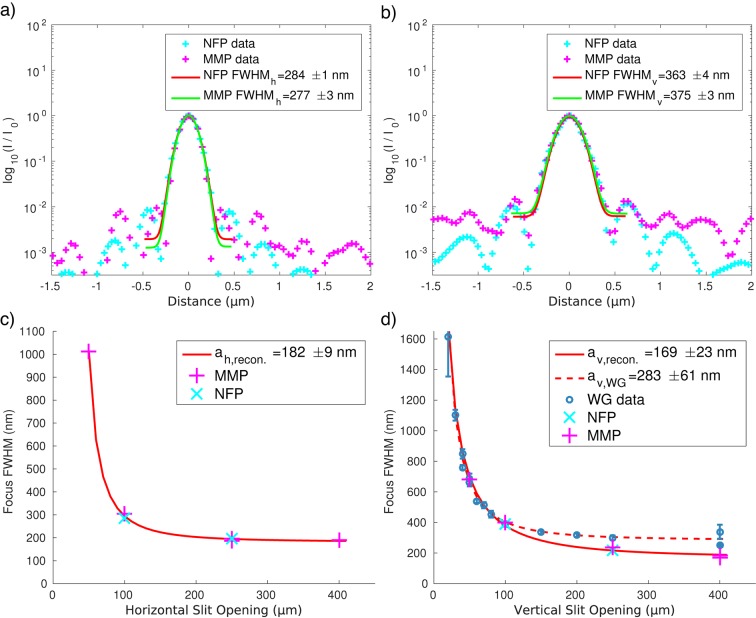
Comparison of reconstructed (normalized) intensity profiles of NFP and MMP in the horizontal (*a*) and vertical (*b*) direction of the focal plane, for the 100 µm × 100 µm slit setting. Using the series of slit settings we illustrate the dependence of focus size on slit opening for the horizontal (*c*) and vertical (*d*) direction. Using the model function equation (9) for the focus width, the size of the source’s image is 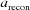 = (182 ± 9) nm × (169 ± 23) nm. (*d*) Same as (*c*) with additional and completely independent results for the focus size, as determined by scanning a waveguide (with entrance size 50 nm).

Next we present the NFP results and a corresponding comparison. A detailed description of NFP can be found in earlier publications (Robisch *et al.*, 2015 ▸; Robisch & Salditt, 2013 ▸). The preprocessing steps for NFP are as follows. The holograms were dark-field corrected. In a next step the holograms have been aligned in the transversal direction *via* a Fourier space registration (Guizar-Sicairos *et al.*, 2008 ▸) using the encoder positions of the scanning motors as a starting guess. For the longitudinal alignment, *i.e.* the correction of propagation distances, an auto-focus algorithm (Langehanenberg *et al.*, 2007 ▸) has been used. The reconstructions were obtained after 25 NFP iterations. The object *O* was initialized with a uniform amplitude of 1 and a phase shift of −0.2 rad. In the first ten iterations the constraint for negative phases has been applied to the object’s guess. *P* was initialized by a back-propagated flat-field. The feedback parameter for *P* was chosen as 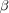 = 0.1 and 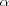 = 0.2 for the object. Note that the necessary Fresnel scaling is applied on the current guess of *O* before projecting on the measurements by resizing the reconstruction matrices. Fig. 3 ▸ presents the results of the NFP reconstruction, for a 100 µm × 100 µm slit opening, and Fig. 4 ▸ shows the results of NFP and MMP in direct comparison, evaluating the reconstructions along the principal axis and the corresponding focal spot sizes for the horizontal (Fig. 4*a* ▸) and vertical (Fig. 4*b* ▸) directions. Good agreement between both completely independent methods is observed, concerning in particular the central peak and the first side oscillation. As a measure for the focus size we use the full width at half-maximum (FWHM) of a Gaussian with linear background fitted to the central peak. Again, we find good agreement between NFP and MMP with (284 ± 1) nm to (277 ± 3) nm horizontally and (363 ± 4) nm to (375 ± 3) nm vertically, respectively. The data acquired for an entire series of slit settings are shown in Figs. 4(*c*) and 4(*d*) ▸, illustrating the dependence of the focus size on the slit opening *d* (numerical aperture), following the expected behaviour. The FWHM curves in Fig. 4(*d*) ▸ are then fitted to (Schroer *et al.*, 2008 ▸)
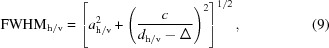
where the first term in the square brackets models the geometric demagnification of the (incoherent) source size 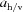 and the second the broadening by diffraction as a function of the slit size 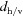 (in front of the KB). Physically, the fitting constant *c* can be related to the focal length and photon energy. Note also that an offset Δ with respect to the nominal slit values was introduced to take into account errors in the calibration of the slit size.

**Figure 5:**
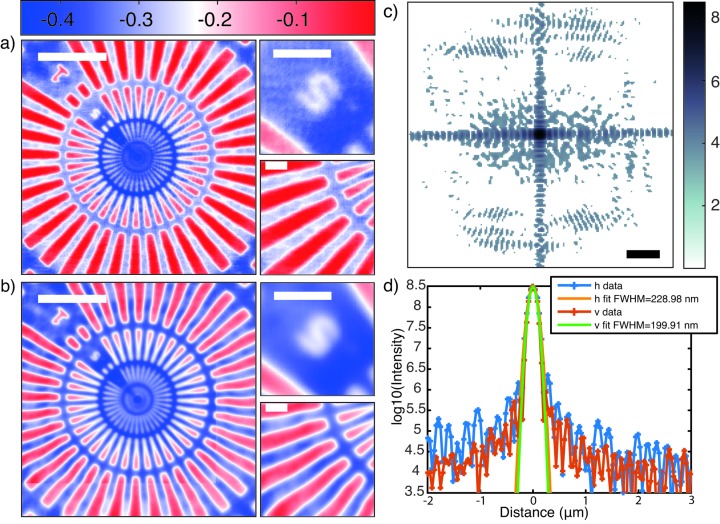
NFP reconstruction of the object, recorded at slit settings of 400 µm × 400 µm (h × v) (beamline exit) and 220 µm × 270 µm (mirror). (*a*) Phases of the NFP reconstruction for the Siemens star pattern. Scale bar: 10 µm. The insets show detail of the 0.5 µm marker (top) and a region of the rays (bottom). Scale bar: 2 µm. (*b*) Reconstruction using the contrast transfer function algorithm (Cloetens *et al.*, 1999 ▸) with the same input as (*a*). Same scale and colour bars as in (*a*). The probe reconstructions corresponding to (*a*) are shown in (*c*), along with (*d*) the corresponding line profiles through the focus. Scale bar for (*c*): 1 µm. Note that the focal width is smaller than in Fig. 4 ▸, owing to the larger slit opening.

Fitting equation (9) to the reconstructed focus sizes yields 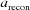 = (182 ± 9) nm × (169 ± 23) nm (h × v). The values for the vertical direction (*d*) are further confirmed by scanning the focal intensity with a waveguide (WG) (Bartels *et al.*, 2015*b* ▸; Neubauer *et al.*, 2014 ▸). From these data we obtain 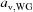 = (283 ± 61) nm. This value is larger due to vibrations of the WG during the scan; also the finite channel width of the WG broadens the intensity distribution. Fig. 5 ▸ presents the results for object reconstruction under optimized illumination (Fig. 5*a* ▸), and also illustrates the benefit in image quality, when compared with a standard object reconstruction (Fig. 5*b* ▸) (without simultaneous probe reconstruction). For this comparison, the contrast transfer function algorithm (Cloetens *et al.*, 1999 ▸) was used, and applied in such a way that the same amount of datasets were used. Both schemes hence have the same set of measurements as input. We clearly observe the benefit of using an iterative algorithm with probe retrieval. Notably, the insets show an improvement in resolution (detail on the 0.5 µm marker) and the removal of some low-frequency image distortions (detail on the rays). We attribute this improvement to the separation of probe and object.

## 5. Summary and outlook   

We have presented a novel approach to reconstructing the extended quasi-spherical wavefront of a hard X-ray nano-probe, in the typical setting of state-of-the-art nanoscale holographic X-ray imaging and tomography using high-gain KB focusing. Importantly, the complex-valued illumination wavefront can be retrieved for the unperturbed case of the actual KB beam, without recourse to additional wavefront modification, as in the scheme given by Stockmar *et al.* (2013 ▸). This goal was accomplished by two completely independent approaches, which differ in the data acquisition scheme and the reconstruction algorithm, namely NFP (Robisch *et al.*, 2015 ▸) and MMP, which we have further adapted and optimized here, with respect to earlier implementations (Allen & Oxley, 2001 ▸; Allen *et al.*, 2001 ▸; Hagemann *et al.*, 2014 ▸). Notably, the current implementation can handle the diverging beam in the laboratory coordinate frame without transformation to equivalent geometries, *e.g.* by application of the Fresnel scaling theorem. While this is clearly more demanding on the computational level, it offers more direct access to the relevant aspects of focusing and propagation without the assumption of a perfect point beam focus. The resulting reconstructions of the NFP and MMP scheme are in very good agreement, and the benefit of (simultaneous) probe retrieval for actual imaging applications was also demonstrated, comparing the superior object reconstructions of NFP (see Fig. 5 ▸) with the standard contrast transfer function approach.

Finally, we point out that this scheme can be extremely useful for the alignment and improvement of the focusing optics. Near-field reconstructions are less sensitive to partial coherence, and even for large slit sizes can give proper information on the probe. However, since the presented method is numerically involved, one may worry about practical procedures which could give fast and robust feedback, for example during beamline alignment. To this end, we show in the supporting information that a simple procedure based on the directly computable autocorrelation function, as computed by fast Fourier transform from the KB far-field, can already help in the optimization of focusing, and when needed can be extended to the full scheme presented here.

## Supplementary Material

Simulated results, raw data for the algorithm and focus characterization via the auto-correlation.. DOI: 10.1107/S160057751700128X/ve5058sup1.pdf

Matlab script of the algorithm.. DOI: 10.1107/S160057751700128X/ve5058sup2.txt

## Figures and Tables

**Table 1:** Experimental parameters for the MMP and NFP recordings

Parameter	MMP	NFP	Unit
Beamline exit slits (h × v)	100 × 100	100 × 100	µm
Mirror slits (h × v)	400 × 500	220 × 270	µm
*z*_01_	–	{80, 85, 90, 94}	mm
*z*	{33.5, 34.4, 35.4, 36.4}	37.2	cm
Pixel size	37	{80, 85, 90, 93}	nm
