# Supplementary material for: Probe reconstruction for holographic X-ray imaging
Source: J Synchrotron Radiat. 2017 Feb 16;24(Pt 2):498–505. doi: 10.1107/S160057751700128X (PMC5330293; doi:10.1107/S160057751700128X)
Supplement: Supplementary file 1 [file s-24-00498-sup1.pdf]

JOURNAL OF SYNCHROTRON RADIATION

Volume 24 (2017)

Supporting information for article:

Probe reconstruction for holographic X-ray imaging

Johannes Hagemann, Anna-Lena Robisch, Markus Osterhoff and Tim Salditt

Supporting Material to Probe Reconstruction for Holographic X-ray Imaging

Johannes Hagemann, Anna-Lena Robisch, Markus Osterhoff, Tim Salditt

November 22, 2016

1 Simulation

We have validated the MMP algorithm by various tests on simulated data. Below we give the example for a Gaussian beam of waist $w_0 = 500\,\mathrm{nm}$ at $8\,\mathrm{keV}$. The simulation parameters are summarized in Tab. 1. Figure 1 (a) and (b) show the simulated beam in a reference plane, chosen at $z = 32\,\mathrm{cm}$, $1\,\mathrm{cm}$ in front of the first detection plane. Next, the beam phases in this plane were deliberately distorted by multiplication with a pure phase image, corresponding to the mandrill shown in (c). Measurements were simulated at distances $z = \{33, 34, 35, 36\}\mathrm{cm}$, with a pixel size of $50\,\mathrm{nm}$. Here it is important to capture the decay of the probe sufficiently well to prevent artifacts in the reconstruction. The cut-off radius in the focal plane was chosen to $q_c = 200 \cdot w_0$. This value is very large, since simulations showed that underestimating the cut-off can lead to artifacts, while choosing it too large does not degrade reconstruction. After convergence of the algorithm, the mandrill image (c) is obtained after division of the curvature term.

Table 1: Simulation parameters.

Parameter	Simulation	Unit
Energy	8	kV
Pixel size	50	nm
z	{33, 34, 35, 36}	cm
Reference plane	32	cm
Fresnel number F^1	{16, 8.1, 5.4, 4}	$\cdot 10^{-4}$
w_0	500	nm
Field of view	205(4096)	µm (px)

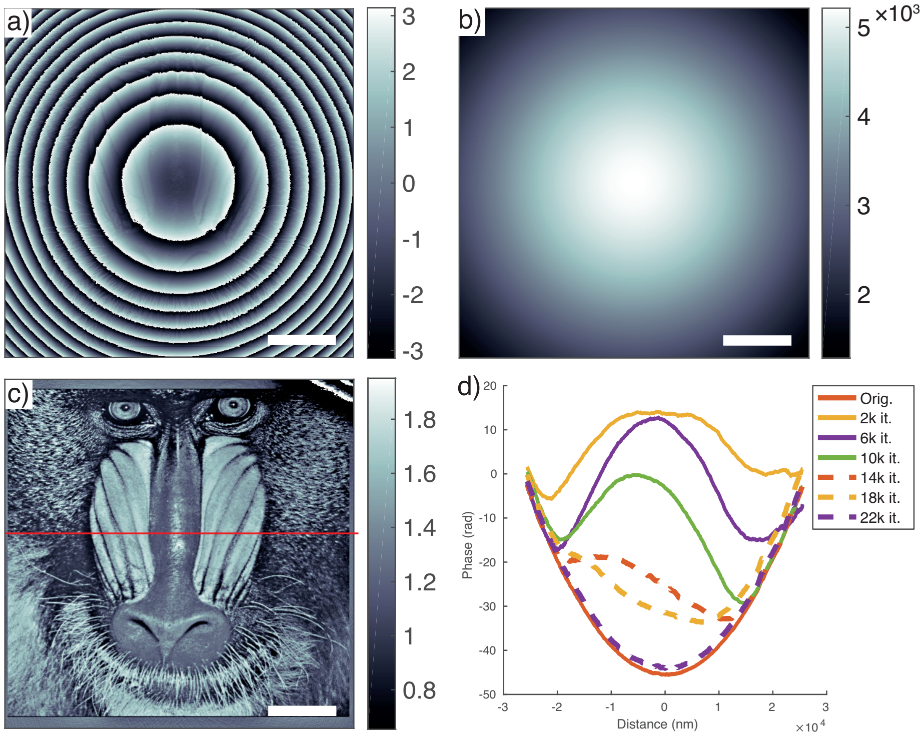

Figure 1: Numerical validation of MMP with a Gaussian beam distorted in the phase by an image of a mandrill (a) and undisturbed amplitudes (b). After convergence of the algorithm, one can extract the mandrill's imprint (c) by removing the curvature. The convergence can be illustrated by plotting line profiles of the phase (d) along the red line indicated in (c). Scale bar indicates 10 µm.

Listing 1 shows the MATLAB implementation of the reconstruction algorithm described in Sec. 3 of the paper. Up to line 62 we allocate memory for error measurements, prepare the propagators(L. 25-30) and support constraint (L. 35-40). From L. 63-104 the actual iteration is carried out with the nested loop over the planes in L. 69

Listing 1: Implementation of sRAAR with support in the focal plane.

```
1 % mmp algorithm with additional projection on focus size
  % constraints = detector measurements
3 % guess = initizialization
  % iterations = # of iterations to do
5 % param = parametar object
  % F_in = set of Fresnel numbers
7 function [reconstruction, errors] = mmp_focus_raar(constraints,
      guess, iterations,...
      param, F_in)
```

```
if param.use_GPU == 1
    constraints = gpuArray(constraints);
    guess = gpuArray(guess);
end

EPSILON = 10 * eps;
%parse arguments
% ... test if param has all setting


%prepare algorithm
h = waitbar(0, 'progress');
waitbar(0, h, ...
    sprintf('Preparing PropagatorGPUs...'));
num_planes = numel(fresnel_num);
% %PropagatorGPUs
for(ii =1:num_planes)
    props{ii} = feval(param.propagator, fresnel_num(ii), fresnel_num(ii), ...
        param.rec_width, param.rec_height,0,1);
end

for(ii =1:num_planes)
    inv_props{ii} = feval(param.propagator, -fresnel_num(ii), -fresnel_num(ii), ...
        param.rec_width, param.rec_height,0,1);
end

% focal plane cut off
d_qx =  (param.z1 * param.lambda)/(param.rec_height*param.pixel_size);
d_qy =  (param.z1 * param.lambda)/(param.rec_width*param.pixel_size);
x = (-param.rec_width/2 : 1 : (param.rec_width/2 - 1)) .* d_qx ;
y = (-param.rec_height/2 : 1 : (param.rec_height/2 - 1)) .* d_qy ;
[X, Y] = meshgrid(x, y);

ind = (sqrt(X.^2 +Y.^2) < param.focus_cut_off);
% smeared out edge
% ind = imgaussfilt(double(ind),1);

if param.use_GPU == 1
    ind = gpuArray(double(ind));
end

%errors
if param.use_GPU == 1
    errors = gpuArray(zeros(iterations, num_planes, 2));
else
    errors = (zeros(iterations, num_planes, 2));
end

b_0 = param.b_0;
b_m = param.b_m;
b_s = param.b_s;

%start iterations
```

```
for ii = 1:iterations
    %     RAAR relaxation
    b = exp(-(ii/b_s)^3)*b_0 + (1 - exp(-(ii/b_s)^3))*b_m;

    waitbar(ii / iterations, h, ...
        sprintf('%d / %d',ii, iterations));

    for jj = 1:num_planes
        guess_old = guess;
        guess = props{jj}.propTF(guess);

        if(isfield(param,'do_errors') == 1)
            if param.do_errors == 1
                tmp = mid(constraints(:,:,jj), param) - ...
                    mid(abs(guess),param);
                errors(ii, jj, 1) = sum(abs(tmp(:)).^2)./ (param.
    height*param.width);
            end
        end

        % project on measurement (Eq. 4)
        guess = MagProj(constraints(:,:,jj), guess, EPSILON);
        guess = inv_props{jj}.propTF(guess);
        P_M = guess;

        % reflect on M
        guess = 2*guess - guess_old;
        R_M = guess;

        %% back in focal plane, projection on S
        focus = fftshift(fft2(ifftshift(abs(guess))));
        focus = focus .* ind;
        focus = fftshift(ifft2(ifftshift(focus)));
        %% recompose phases in sample plane
        guess = abs(focus) .* exp(1i*(angle(focus) +angle(guess)));

        % reflect on S
        guess = 2*guess - R_M;

        % new iterate (Eq. 1)
        guess = (b/2) * (guess + guess_old) + (1 -b)*P_M;
    end %planes
end % iterations

for jj = 1:num_planes
    tmp = props{jj}.propTF(guess);
    figure
    imagesc(abs(tmp).^2)
    sum(abs(tmp(:)))
    title(sprintf('reconstructed wavefield at %f', param.
    det_distances(jj)))
end
% get results back to host
reconstruction = gather(guess);
errors = gather(errors);
close(h);
end
```

2 Fitted Parameters for Source Size

Table 2 summarizes the fitted parameters for Eq. 9 of the source size fit.

Table 2: Fitted parameters for source size (cf. Figure 4).

Parameter	$a_{\text{h, recon.}}$	$a_{\text{v, recon.}}$	$a_{\text{v, WG}}$
a (nm)	182(9)	169(23)	238(61)
c (m^2)	$1.5 \cdot 10^{-11}$	$3.4 \cdot 10^{-11}$	$2.75 \cdot 10^{-11}$
Δ (µm)	34.8(3)	0	38(3)

3 Raw Data

Figure 2 and 3 show exemplary raw data for the MMP scheme. Recorded with a scintillator(LUAG) coupled PCO.2000 detector using a 20x microscope objective lens, resulting in 370 nm pixel size.

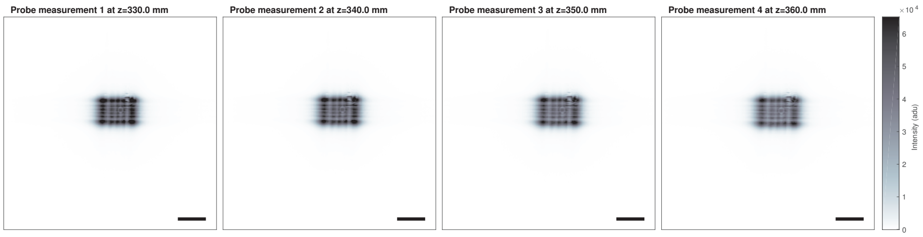

Figure 2: Darkfield corrected probe measurements for MMP for 100 µm × 100 µm slit opening. Scale bar indicates 100 µm.

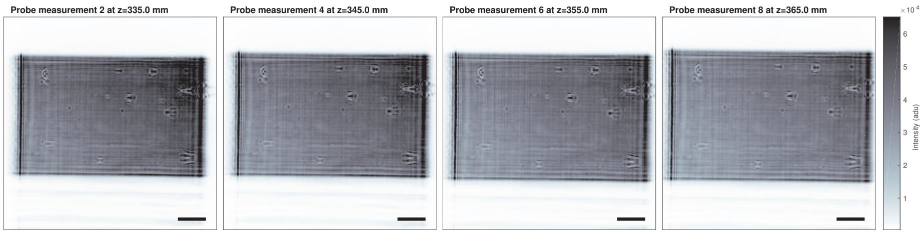

Figure 3: Darkfield corrected probe measurements for MMP for 400 µm × 400 µm slit opening. Scale bar indicates 100 µm.

4 Focus Width from Autocorrelation

Since the presented methods are numerically involved, one may worry about practical procedures which could give fast and robust feedback, for example

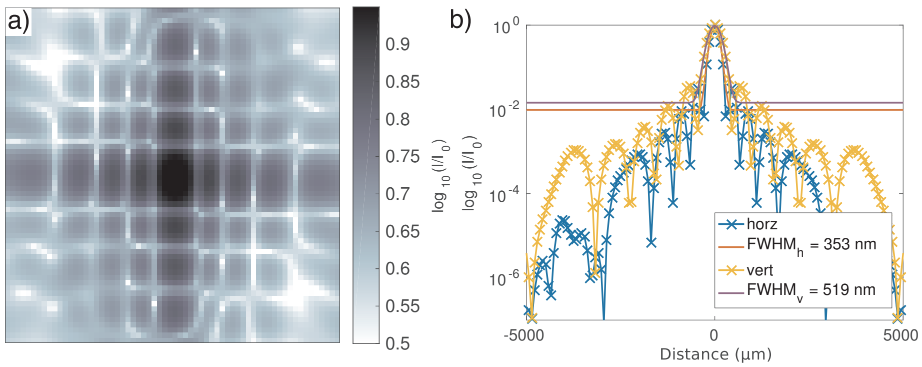

Figure 4: Exemplary calculation of the autocorrelation (AC) function, for $100 \times 100\,\mu\mathrm{m}^2$ slit setting obtained from intensity measurement. (a) modulus of the 2D AC in the focal plane. (b) line profiles of the AC function along horizontal and vertical direction.

during beamline alignment. The presented reconstruction schemes both require extensive numerical calculations and are thus not suited for an online alignment of the KB-mirrors. Here we want to answer if the focus size can also be estimated from a simple empty beam measurement. Figure 4 shows (a) the autocorrelation (AC) function calculated from the intensity measurement and (b) Gaussian line fits of the profiles extracted from (a). Comparing the resulting FWHMs with the results from Fig. 4 of the paper we note a factor of 1.27 (h) and 1.38 (v) between the widths. From simulations we deduced a factor of $1.41 \approx \sqrt{2}$ between the squared modulus FWHM of the AC and focus intensity FWHM, for the undisturbed Gaussian beam. Introducing a phase modulation in the beam as described in Sec. 1 yields a slightly different factor for the ratio $\mathrm{FWHM}_{\mathrm{AC}}/\mathrm{FWHM}_{\mathrm{focus}} = 1.35$. The AC can already help in the optimization of focusing, and when needed can be extended to the full scheme presented here.
